# The Impact of Sarcopenia on Clinical Outcomes in Pediatric Crohn’s Disease

**DOI:** 10.1093/ibd/izaf193

**Published:** 2025-09-05

**Authors:** Giulia D’Arcangelo, Delia De Mitri, Ludovica Busato, Lorenza Bottino, Francesca Maccioni, Andrea Verrino, Marina Aloi

**Affiliations:** Pediatric Gastroenterology and Liver Unit, Sapienza University of Rome, Rome, Italy; Pediatric Gastroenterology, Hepatology and Cystic Fibrosis Unit, Fondazione IRCCS Cà Granda, Ospedale Maggiore Policlinico di Milano, Milan, Italy; Pediatric Gastroenterology and Liver Unit, Sapienza University of Rome, Rome, Italy; Department of Radiology, Sapienza University of Rome, Rome, Italy; Department of Radiology, Sapienza University of Rome, Rome, Italy; Department of Radiology, Sapienza University of Rome, Rome, Italy; Pediatric Gastroenterology and Liver Unit, Sapienza University of Rome, Rome, Italy; Pediatric Gastroenterology, Hepatology and Cystic Fibrosis Unit, Fondazione IRCCS Cà Granda, Ospedale Maggiore Policlinico di Milano, Milan, Italy; Department of Pathophysiology and Transplantation, Università degli Studi di Milano, Milan, Italy

**Keywords:** children, muscle mass, Crohn's disease, body composition

## Abstract

**Background:**

The effect of sarcopenia on clinical outcomes in children with Crohn’s disease (CD) is unknown. We investigated whether sarcopenia at the diagnosis impacts the outcomes of children with CD.

**Methods:**

This was a retrospective, single-center, case-control study of newly diagnosed children with CD undergoing magnetic resonance (MR) within 1 month from the diagnosis, from 2011 to 2022. Sarcopenia was assessed by measuring total psoas muscle area (tPMA) at L3-L4 level on the MR and defined as *z*-score values ≤2 SDs. Children with and without sarcopenia were compared for the risk of disease flares, CD-related hospitalization, complications, need for step-up treatment, and courses of steroids over a 2-year follow-up.

**Results:**

Seventy-eight children were included (median age 10.7 years), 46 (59%) with sarcopenia and 32 (41%) without. The risk of clinical relapse was higher in patients with sarcopenia at 6 [19.5% vs 3%, odds ratio (OR) 7.5 (95% CI, 1.5-85)] and 12 months [30% vs 6%, OR 6.5 (95% CI, 1.4-30.4)]. Kaplan-Meier analysis showed lower survival free from relapses in children with sarcopenia (log rank *P* = .01, hazard ratio 2.7, 95% CI, 1.4-4.5). Multivariate analysis identified sarcopenia as independent predictors of clinical relapses (OR 1.7, 95% CI, 1-3.1, *P* = .045). No other independent predictor of unfavorable outcome was detected.

**Conclusions:**

The presence of sarcopenia at the diagnosis increases the risk of clinical relapses in the first year of diagnosis. Magnetic resonance evaluation of the tPMA could therefore help identify children at higher risk of worse outcomes.

Key messages

- **What is already known?** Pediatric Crohn’s disease (CD) increases malnutrition risk, affecting 16%-87% of cases and leading to sarcopenia—a loss of muscle mass and strength. Sarcopenia is poorly studied in pediatric CD.
- **What is new here?** In our cohort, 59% of children had sarcopenia (more than previously reported). Sarcopenia was linked to increased relapse risk, particularly within 6 months, and identified as an independent predictor of relapse.
- **How can this study help patient care?** As a negative prognostic marker of disease course, addressing sarcopenia might improve outcomes. Future research should focus on body composition’s role in disease progression and establishing protocols to enhance clinical outcomes and quality of life.

## Introduction

Pediatric Crohn’s disease (CD) is associated with an increased risk of nutrient malabsorption,^1–3^ which, along with the catabolic state induced by chronic inflammation, reduced food intake, and active loss of nutrients,^4^ can lead to malnutrition, which is characterized by protein–energy and micronutrient deficiencies (16%-87% of cases), weight loss (70% of cases) and growth retardation (up to 40% of cases).^5–7^

Malnutrition is a risk factor for worse outcomes^8^ and can determine changes in body composition that lead to sarcopenia. Sarcopenia was first described in 1989 by Rosenberg as the loss of skeletal muscle mass and strength associated with physical disability and poor quality of life.^9^ The International Consensus on Sarcopenia defined sarcopenia as a skeletal muscle index (SMI) 2 SDs below the norm for young, healthy adults,^10^ consequently reducing muscle strength or physical performance.

Different imaging and non-imaging modalities are used to assess body composition and muscle mass. Non-imaging techniques include air displacement plethysmography, hydrostatic weighing, anthropometric measures such as mid-arm circumference combined with triceps skinfold thickness, and bioelectrical impedance analysis (BIA). Imaging techniques include dual-energy X-ray absorptiometry (DXA), dilution techniques, computed tomography, and magnetic resonance imaging (MRI).^11^^,^^12^ Magnetic resonance imaging, in particular, is routinely used in children with CD at diagnosis and during follow-up^13^ and may therefore represent the tool of choice to assess body composition and SMI in this setting.

The impact of sarcopenia has been investigated in various chronic conditions in adults, including inflammatory bowel disease (IBD), and it has been associated with worse outcomes (eg, postoperative complications).^14–17^ A reduced muscle mass (measured by MRI) was found in pediatric patients with IBD compared to healthy controls, and sarcopenia was proven to be a risk factor for biological therapy and disease exacerbation.^18^ Nonetheless, pediatric data remain scarce and hindered by the lack of consensus on the definition of sarcopenia in this setting.

We therefore aimed to determine the impact of sarcopenia at the diagnosis on clinical outcomes in children with CD. Specifically, we evaluated^1^ the impact of sarcopenia on disease progression by comparing risk of relapse, hospitalization, disease-related complications, therapeutic step-up, and corticosteroid use between children with and without sarcopenia and analyzed associated risk factors for the same outcomes^2^; we compared clinical and laboratory parameters in both groups at 6, 12, and 24 months and conducted a subgroup analysis evaluating the resolution rate of sarcopenia identifying risk factors associated with its persistence.

## Materials and methods

### Study design and inclusion criteria

This longitudinal, retrospective case-control study was conducted at the Pediatric Gastroenterology and Hepatology Unit of Umberto I Hospital in Rome (Sapienza University). Medical records of all patients under 18 years diagnosed with CD^13^ between 2009 and 2023 who underwent MRI at diagnosis were analyzed. Individuals were only included if they had cross-­sectional abdominal imaging via magnetic resonance (MR) scan within 30 days of diagnosis.

### Data collection

Medical records and radiological MR images were retrospectively reviewed. Data were collected from the diagnosis up to 24-month follow-up and included demographic (age at the diagnosis, gender, and family history), anthropometric [including weight, height, body mass index, and relative age and gender *z*-scores], and laboratory data [hemoglobin, red and white blood cells, platelets, lipid profile [total cholesterol, low-density lipoprotein (LDL), high-density lipoprotein (HDL), triglycerides], iron profile (serum iron, ferritin), liver function markers (aspartate aminotransferase and alanine aminotransferase gamma-glutamyl transferase), inflammatory markers (erythrocyte sedimentation rate [ESR] and C-reactive protein [CRP]), fecal calprotectin, and serum levels of total proteins, albumin, creatinine and creatine phosphokinase (CPK)].

Disease activity was evaluated using the weighted Pediatric Crohn’s Disease Activity Index (wPCDAI),^19^ and mucosal inflammation was assessed with the Simple Endoscopic Score for Crohn’s Disease (SES-CD) following endoscopic exam reviews.^20^

Disease localization, extent, and behavior were defined using the Paris classification.^21^

At 6, 12, and 24 months, data on outcomes were collected, including disease relapses, defined as a wPCDAI score > 12.5 points after the achievement of remission, or an increase of 20 points from the previous assessment; number of disease complications (strictures and/or fistulas); unplanned hospitalizations related to a disease relapse; therapeutic escalations defined as the need of escalating to a biologic therapy or to a second-line biologic or small molecule when already under anti-­tumor necrosis factor (anti-TNF) agents; and number of systemic corticosteroid courses.

### MRI and definition of sarcopenia

Routine MRs performed at diagnosis and during follow-ups were analyzed. Two independent reviewers (F.M. and L.B.), blinded to other data, calculated the total psoas muscle area (tPMA) at the L3-L4 intervertebral disc levels by summing both sides to obtain the tPMA in mm^2^. Magnetic resonance images were retrieved from the hospital’s Picture Archiving and Communication System. Examinations were performed using either a 1.5 T (Siemens MAGNETOM Avanto 1.5 T; Siemens Healthineers) or 3 T (Discovery MR750 3 T; GE HealthCare) magnet. The psoas muscle area measurements were obtained on axial T2-weighted images without fat suppression, using HASTE or SSFSE sequences, and the appropriate intervertebral disc level was identified with a pointer function based on coronal T2-weighted images. Patients were evaluated for sarcopenia based on MRE data using an online tool for pediatric reference values (https://ahrc-apps.shinyapps.io/sarcopenia) and based on the gender- and age-specific *z*-scores for tPMA at L3-4 levels as has been previously validated and used.^14^^,^^16^^,^^22^^,^^23^ Values with a *z*-score < −2.00 SD were defined as sarcopenia.

### Statistical analysis

Descriptive statistics were presented as median (IQR) for continuous variables and frequencies (%) for categorical variables. Univariable comparisons were performed using chi-squared or Fisher’s exact tests for categorical variables and a Wilcoxon rank-sum test for continuous variables. An intention-to-treat approach was used to analyze all eligible participants based on their baseline classification. Survival analyses compared the time-to-event outcomes between patients with and without sarcopenia. Kaplan-Meier survival curves were generated, and differences between the groups were assessed using the log-rank test. Hazard ratios (HRs) and their corresponding 95% CIs were estimated using Cox proportional hazards regression to account for potential confounders. Univariate and multivariate logistic regression models were applied to assess the association between the presence of sarcopenia and disease outcomes. For the multivariate model, we employed a stepwise approach, incorporating variables that were either statistically significant in univariate analysis (*P* < .01) or deemed clinically relevant in influencing the outcome of interest. Given the need to balance model complexity and stability while avoiding overfitting, we adhered to the rule of including no more than one independent variable per 10 observed outcome events. In cases where the number of events limited model capacity, we prioritized clinically relevant variables over statistically significant but biologically less meaningful predictors. Results were presented as odds ratios (ORs) with 95% CIs. All statistical analyses were 2-tailed with the significance threshold set at *P* < .05. Data were analyzed using GraphPad Prism (GraphPad Prism 9.5.1.733) of Windows, GraphPad software. A post hoc power analysis was performed to assess the adequacy of the sample size for detecting differences in disease relapse rates between patients with and without sarcopenia (65% vs 28%). Using a 2-group comparison of proportions at a significance level of 0.05, the estimated statistical power was 66%, indicating moderate power but below the conventional 80% threshold. Our sample size aligns with prior retrospective studies on sarcopenia in pediatric CD.^16^^,^^18^

## Results

### Participant characteristics by study group

Seventy-eight CD patients were included, with a median age of 12 years (IQR 9-15) at the diagnosis. Based on MR evaluations, 46 patients (59%) were diagnosed with sarcopenia, and 32 (41%) without. The median tPMA measured in patients with sarcopenia was 994 mm^2^ (835-1373) at the L3-L4 level, whereas it was 1858 mm^2^ (1615-2267) in those without (*P* < .0001). The median *z*-score of tPMA at L3-L4 was −2.09 (−2.8 to 2) in the group with and −0.8 (−1.2 to −0.23) in the group without sarcopenia (*P* < .0001).

Table 1 reports the differences between the 2 groups regarding clinical, laboratory, and disease characteristics at diagnosis. Patients with sarcopenia more frequently had L3 disease location (87% vs 59%, *P *= .007) and higher endoscopic inflammation (SES-CD: 15 vs 11, *P *= .04). Conversely, they never presented with an isolated ileal (L1) disease (0% vs 34%, *P* < .001). Moreover, they more often received anti-TNF agents as induction (52% vs 31%, *P *= .1), and this was in all cases administered to patients with extensive disease.

**Table 1.** Baseline characteristics of children with and without sarcopenia at the diagnosis.

Characteristics of the population	Children with sarcopenia *n* = 46 (59%)	Children without sarcopenia *n* = 32 (41%)	*P*
Age, *N* (%)			
*1-10*	20 (44)	9 (28)	.2
*11-18*	26 (56)	23 (72)	
Age, median (IQR)	11.1 (8-13,2)	11.5 (7.2-14)	.9
Weight *z*-score, median (IQR)	−0.3 (−0.9-0,1)	0.1 (−0.2-0.8)	.07
BMI *z*-Score, *N* (%)			
*<−2 DS*	2 (4)	0 (0)	0.5
*>−2 DS*	44 (96)	32 (100)	
Sex, *N* (%)			.1
*M*	24 (52)	22 (69)	
*F*	22 (48)	10 (31)	
Location, *N* (%)			
*L1*	0 (0)	11 (34)	<.001
*L2*	2 (4)	0 (0)	.5
*L3*	40 (87)	19 (59)	.007
*L4*	26 (57)	12 (37)	.11
*P*	16 (35)	15 (47)	.3
Behavior, *N* (%)			
*B1*	40 (87)	24 (75)	.2
*B2*	4 (9)	8 (25)	.06
*B3*	2 (4)	2 (6)	1
wPCDAI, median (IQR)	37.5 (18.7-45)	31.25 (25.6-31.2)	.4
MINI index, median (IQR)	9.5 (5.2-18)	9 (9-11.7)	.7
SESCD, median (IQR)	15 (13-20)	11 (9.2-17)	.04
tPMA (mm^2^), median (IQR)	994 (835-1373)	1858 (1615-2267)	<.0001
Protein (g/L), median (IQR)	72 (70-76)	65.5 (58-72)	.002
Protein < 64 g/L	5 (11)	8 (25)	.12
Albumin (g/L), median (IQR)	39.5 (36.5-42)	36.5 (33-38)	.02
Albumin < 35 g/L	8 (17)	8 (25)	.56
CPK (U/L), median (IQR)	33.5 (25-61)	52.4 (27-87)	.1
CPK < 30 U7L	5 (11)	0 (0)	.07
CRP (mg/dL), median (IQR)	1.4 (0.7-3.2)	2.8 (0.6-5.1)	.2
CRP > 0.5 mg/dL	30 (65)	16 (50)	.24
ESR (mm/h), median (IQR)	58 (38-85)	51 (32-69)	.2
ESR > 25 mm/h	36 (78)	18 (56)	.04
Hb (g/dL), median (IQR)	11.8 (10.5-13)	12.1 (11.2-12.5)	.8
Anemia^a^	23 (50)	10 (31)	.11
Total cholesterol, (mg/dL), median (IQR)	138.5 (119-164)	92 (80-135)	.009
LDL cholesterol, (mg/dL), median (IQR)	80.5 (57-86)	47 (42-84)	.03
HDL cholesterol, (mg/dL), median (IQR)	43 (35.7-45.5)	38 (33-41)	.06
Triglycerides (mg/dL), median (IQR)	97 (68-130)	66 (60-96)	.1
Calprotectin (µg/mL), median (IQR)	227 (80-771)	280 (83-543)	.6
Induction treatment, *n* (%)			
Nutritional therapy (EEN or diet + PEN)	4 (9)	8 (25)	.06
CS	19 (41)	15 (47)	.64
Anti-TNF	24 (52)	10 (31)	.1
Maintenance treatment, *n* (%)			
Nutritional therapy (EEN or diet + PEN)	2 (4)	3 (9)	.39
Anti-TNF	33 (72)	19 (59)	.33
IM	13 (28)	11 (34)	.62

a Based on gender and age.

Abbreviations: anti-TNF, anti-tumor necrosis factor; BMI, body mass index; CPK, creatine phosphokinase; CRP, C-reactive protein; CS, corticosteroids; EEN, exclusive enteral nutrition; ESR, erythrocyte sedimentation rate; Hb, hemoglobin; HDL, high-density lipoprotein; IM, immunomodulator; IQR, interquartile range; LDL, low-density lipoprotein; PEN, partial enteral nutrition; SES-CD, Simple Endoscopic Score for Crohn’s disease; wPCDAI, weighted Pediatric Crohn’s Disease Activity Index.

### Risk of adverse outcomes in children with and without sarcopenia

The analysis of the risk of adverse outcomes in the 2 at 6, 12, and 24 months is reported in Table 2. Patients with sarcopenia were at higher risk of relapses at 6 months [19.5% vs 3%, OR 7.5 (95% CI, 1.5-85), *P* = .04] and 12 months [28% vs 6%, OR 5.9 (95% CI, 1.2-27), *P* = .01]. No other significant differences were detected for the other outcomes at any time point during follow-up.

**Table 2.** Risk of adverse outcomes in children with and without sarcopenia at the diagnosis at 6, 12, and 24 months.

	Children with sarcopenia	Children without sarcopenia		
Outcome	*N* = 46	*N* = 32	*P* value	OR (95% CIs)
**6 months**				
Relapses, *n* (%)	9 (19.5)	1 (3)	.04	7.5 (1.5-85)
Hospitalizations, *n* (%)	5 (11)	1 (3)	.2	3.7 (0.4-45.8)
Therapy escalation, *n* (%)	8 (17)	4 (12,5)	.7	1.4 (0.4-4.7)
Complications, *n* (%)	2 (4)	1 (3)	1	1.4 (0.1-21)
CS courses, *n* (%)	8 (17)	6 (19)	1	0.9 (0.3-3.1)
**12 months**				
Relapses, *n* (%)	13 (28)	2 (6)	.01	5.9 (1.2-27)
Hospitalizations, *n* (%)	8 (17)	2 (6)	.18	3.1 (0.6-15)
Therapy escalation, *n* (%)	11 (24)	7 (22)	.95	1.03 (0.4-3)
Complications, *n* (%)	3 (6.5)	2 (6)	.99	1.04 (0.2-6.1)
CS courses, *n* (%)	8 (17)	4 (12.5)	.75	1.77 (0.4-4.7)
**24 months**				
Relapses, *n* (%)	8 (17)	6 (19)	.58	0.91 (0.3-3.1)
Hospitalizations, *n* (%)	5 (11)	5 (16)	.73	0.65 (0.1-2.3)
Therapy escalation, *n* (%)	10 (22)	6 (19)	.79	1.2 (0.4-3.9)
Complications, *n* (%)	2 (4)	1 (3)	1	1.4 (0.1-21)
CS courses, *n* (%)	8 (17)	4 (12.5)	.75	1.4 (0.4-4.7)

Abbreviations: CIs, confidence intervals; CS, corticosteroid; OR, odds ratio.

**Figure 1.**
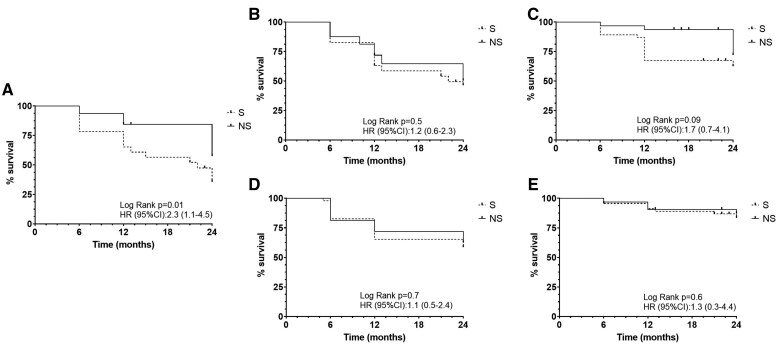
Kaplan-Meier survival analysis comparing outcomes in children with and without sarcopenia. The figure illustrates time-to-event survival curves for 5 clinical outcomes in pediatric patients, stratified by sarcopenia status (presence vs absence). Log-rank tests were used to assess statistical significance between groups (*P* < .05 considered significant). (A) Clinical relapses; (B) treatment escalation; (C) hospitalization; (D) steroid course; (E) complications.

Survival free from clinical relapse was lower in children with sarcopenia (log rank *P* = .01, HR 2.7, 95% CI 1.4-4.5) (Figure 1A), while Kaplan-Meier survival analysis revealed no significant differences between the 2 groups for the remaining outcomes, as indicated by log-rank test results (*P* > .05 for all comparisons) (Figure 1B-E).

### Univariable and multivariable risk factors for all the outcomes

The analysis of risk factors for all the outcomes is reported in Table 3. At the multivariable logistic regression analysis, sarcopenia emerged as a significant independent predictor of disease relapse in both univariate (OR 4.6, 95% CI, 1.7-13.2, *P *= .002) and multivariate analyses (OR 1.7, 95% CI, 1-3.1, *P *= .04), with strong model performance [Area under the receiver operating characteristics (AUROC) 0.87, *P *< .0001]. For hospitalization and complications, no significant predictors were identified. The predictive value of biomarkers (CRP, albumin) and disease activity scores (wPCDAI, SES-CD) was limited across all outcomes.

**Table 3.** Logistic regression analysis of selected possible predictive factors of relapse, hospitalization, complications, treatment escalation, and steroid courses.

Relapse	Univariate analysis		Multivariate analysis	
	Or (95% CI)	*P*-value	Or (95% CI)	*P*-value
Sarcopenia	4.6 (1.7-13.2)	.002	1.7 (1-3.1)	.04
Albumin < 35 g/L	0.96 (0.37-1.8)	.33	4.3 (0.1.27)	.4
CRP > 0.5 mg/dL	1.12 (0.4-3.4)	.83	0.65 (0.05-5)	.7
ESR > 25 mm/h	5.17 (0.9.-17)	.08	3.4 (0.16-156)	.4
wPCDAI	0.9 (0.9-1.02)	.8	0.9 (0.9-1.05)	.7
SES CD	0.9 (0.8-1.07)	.5	1.1 (0.9-1.4)	.1
TNF upfront	0.7 (0.2-1.8)	.4	4 (0.9-47)	.08
*z*-score L4-L5 tPMA	0.6 (0.2-1.3)	.2	4.4 (0.7-9)	.19
**Area under the ROC curve** Area 0.87 SE 0.05 95% CI, 0.7-0.9 *P* value < .0001 NPV (%) 78.2 PPV (%) 83.3 Pseudo R squared 0.27				

Hospitalization	Univariate analysis		Multivariate analysis	
	OR (95% CI)	*P*-value	OR (95% CI)	*P*-value
Sarcopenia	1.6 (0.6-4)	.3	1.6 (0.4-13)	0.14
Albumin < 35 g/L	1.3 (0.4-4.6)	.5		
CRP > 5 mg/dL	1.3 (0.4-4.1)	.6		
ESR > 25 mm/h	0.14 (0.09-0.87)	.07	0.9 (0.01-1.3)	0.08
wPCDAI	0.9 (0.9-1.0)	.1		
SES CD	1 (0.89-1.1)	.8		
TNF upfront	0.8 (0.2-2.1)	.6		
*z*-score L4-L5 tPMA	1 (0.4-2)	.9	1.03 (0.28-3.9)	0.95
**Area under the ROC curve** Area 0.63 SE 0.08 95% CI, 0.46-0.79 *P* value .12 NPV (%) 56.7 PPV (%) 87.5 Pseudo R squared 0.11				

Complications	Univariate analysis		Multivariate analysis	
	OR (95% CI)	*P*-value	OR (95% CI)	*P*-value
Sarcopenia	0.9 (0.3-2.4)	.9		
Albumin < 35 g/L	1.05 (0.01-1.2)	.1		
CRP > 0.5 mg/dL	2.3 (.05-16.4)	.3		
ESR > 25 mm/h	2.1 (0.3-41)	.4		
wPCDAI	0.9 (0.4-2.4)	.4		
SES CD	0.9 (0.8-1.03)	.1		
TNF upfront	1.3 (0.4-3.6)	.5		
*z*-score L4-L5 tPMA	0.9 (0.4-1.8)	.7		

Treatment escalation	Univariate analysis		Multivariate analysis	
	OR (95% CI)	*P*-value	OR (95% CI)	*P*-value
Sarcopenia	1.6 (0.6-4.2)	.2	3.7 (0.1-116)	.4
Albumin < 35 g/L	1.2 (0.3-4.3)	.6	0.2 (0.1-3.4)	.28
CRP > 0.5 mg/dL	1.4 (0.4-4.5)	.5		
ESR mm/h	0.3 (0.04-1.4)	.1	0.04 (0.06-12)	.96
wPCDAI	1 (0.9-1.03)	.7		
SES CD	1.01 (0.9-1.1)	.8		
TNF upfront	0.2 (0.07-0.7)	.01		
*z*-score L4-L5 tPMA	1.1 (0.5-2.3)	.7	2.1 (0.4-80)	.35
**Area under the ROC curve** Area 0.69 SE 0.08 95% CI, 0.5-0.8 *P* value.03 NPV (%) 63.6 PPV (%) 57.9 Pseudo R squared 0.13				

Steroid courses	Univariate analysis		Multivariate analysis	
	OR (95% CI)	*P*-value	OR (95% CI)	*P*-value
Sarcopenia	0.6 (0.2-1.2)	.001	0.64 (0.2-2.03)	.15
Albumin < 35 g/L	0.5 (0.1-1.7)	.3	0.16 (0.07-1.5)	.13
CRP > 0.5 mg/dL	1.5 -70.4-5.5)	.4		
ESR mm/h	0.3 (0.06-1.4)	.1		
wPCDAI	2.2 (0.7-6.9)	.5		
SES CD	0.4 (0.05-2.6)	.1	1.06 (0.9-1.2)	.42
TNF upfront	1.6 (0.7-3.6)	.7		
*z*-score L4-L5 tPMA	0.7 (0.1-3.2)	1.3	0.09 (0.02-1.4)	.14
**Area under the ROC curve** Area 0.69 SE 0.08 95% CI, 0.52-0.85 *P* value.03 NPV (%) 65.9 PPV (%) 69.7 R squared 0.11				

Abbreviations: CI, confidence interval; CRP, C-reactive protein; ESR, erythrocyte sedimentation rate; NPV, negative predictive value; OR, odds ratio; PPV, positive predictive value; ROC, receiver operating characteristics; SE, standard error; SES-CD, Simple Endoscopic Score for Crohn’s disease; tPMS, total psoas muscle area; wPCDAI, weighted Pediatric Crohn’s Disease Activity Index.

### Laboratory and clinical parameters at follow-up

The comparison of the laboratory parameters of disease activity and lipid profile in cases and control during follow-up is shown in Table 4. Alteration of the lipid profile persisted at 6 months follow-up in children with sarcopenia having higher total cholesterol and LDL levels compared to controls [142 (122.5-157.8) mg/dL vs 92 (80-135) mg/dL, *P* = .0001 and 78.5 (66-92) mg/dL vs 47 (42-84) mg/dL, *P* = .001, respectively]. Inflammatory markers, as well as wPCDAI, were higher in cases compared to controls [CRP 1.87 mg/dL (0.06-3.07) vs 0.81 mg/dL (0.37-1.09), *P* = .009 and ESR 51 mm/h (32-69) vs 22 mm/h (16-38), *P* = .01, wPCDAI 21 (15.6-38.2) vs 15 (0-22.5), *P* = .04]. These differences were no longer observed at 12 and 24 months.

**Table 4.** Comparison of the laboratory values at 6-, 12-, and 24-month follow-up between children with and without sarcopenia.

	Children with sarcopenia	Children without sarcopenia	*P*
6 months			
BMI *z*-score, median (IQR)	−0.02 (−0.26 to 1.04)	0.45 (−0.02 to 0.85)	.07
Protein (g/L), median (IQR)	71 (63-77)	67.5 (58-72)	.059
Albumin (g/L), median (IQR)	43 (42-44)	38 (36-41)	.2
CRP (mg/dL), median (IQR)	1.87 (0.06-3.07)	0.81 (0.37-1.09)	.009
ESR (mm/h), median (IQR)	51 (32-69)	22 (16-38)	.01
TOT cholest. (mg/dL), median (IQR)	142 (122.5-158)	92 (80-135)	.0001
LDL cholest. (mg/dL), median (IQR)	78.5 (66-92)	47 (42-84)	.001
HDL cholest. (mg/dL), median (IQR)	43 (34.5-55.7)	48 (39-65)	.09
Triglycerides(mg/dL), median (IQR)	91.5 (72-116.5)	66 (60-96)	.2
wPCDAI, median (IQR)	21 (15.6-38.2)	15 (0-22.5)	.04
MINI index, median (IQR)	9.5 (5.25-18)	9 (9-11.75)	.7
12 months			
BMI *z*-score, median (IQR)	0.57 (−0.12 to 1.1)	0.69 (0.08-1.2)	.5
Protein (g/L), median (IQR)	75 (71-78)	74 (70-75)	.9
Albumin (g/L), median (IQR)	46 (41-48)	46 (44-48.7)	.1
CRP (mg/dL), median (IQR)	0.26 (0.09-1.17)	0.1 (0.06-0.37)	.3
ESR (mm/h), median (IQR)	27 (7.7-35.2)	9 (3.7-18.7)	.01
TOT cholest. (mg/dL), median (IQR)	139.5 (125-167)	130 (106-157.5)	.1
LDL cholest. (mg/dL), median (IQR)	81 (69-99)	74.5 (54.2-100.8)	.3
HDL cholest. (mg/dL), median (IQR)	47 (37.5-57.2)	45 (40.7-52.5)	.6
Triglycerides(mg/dL), median (IQR)	85.5 (65.2-116)	66 (53.5-106.8)	.3
wPCDAI, median (IQR)	15 (0-30)	7.5 (0-10)	.1
MINI index, median (IQR)	0.5 (0-5)	0.5 (−3 to 5)	.6
24 months			
BMI *z*-score, median (IQR)	0.5 (−0.1 to 1.02)	0.69 (0.08-1.28)	.4
Protein (g/L), median (IQR)	77 (71.7-79.5)	74 (70-75)	.4
Albumin (g/L), median (IQR)	46 (44-48)	46 (44-48.7)	.9
CRP (mg/dL), median (IQR)	0.13 (0.06-0.5)	0.1 (0.06-0.3)	.8
ESR (mm/h), median (IQR)	18 (12-24)	9 (3.7-18.7)	.07
TOT cholest. (mg/dL), median (IQR)	133 (126.5-155)	130 (106-157.5)	.4
LDL cholest. (mg/dL), median (IQR)	74 (66.7-83)	74.5 (54.2-100.8)	.5
HDL cholest. (mg/dL), median (IQR)	47 (44.5-53.5)	45 (40.7-52.5)	.2
Triglycerides(mg/dL), median (IQR)	66 (45-89)	66 (53.5-106.8)	.2
wPCDAI, median (IQR)	10 (5-17.5)	7.5 (0-10)	.7
MINI index, median (IQR)	0 (−2.75 to 1.75)	0.5 (−3 to 5)	.5

Abbreviations: BMI, body mass index; CRP, C-reactive protein; DS, standard deviation; ESR, erythrocyte sedimentation rate; HDL, high-density lipoprotein; IQR, interquartile range; LDL, low-density lipoprotein; wPCDAI, weighted Pediatric Crohn’s Disease Activity Index.

### Subgroup analysis: persistence of sarcopenia and risk factors for persistence

A total of 38 patients with sarcopenia at the diagnosis underwent MR reevaluation at 24 months, and among them, sarcopenia had resolved in 18/38 patients (39%). Twenty-three of the 32 children without baseline sarcopenia had an MR performed at 24-month follow-up: no cases of new-onset sarcopenia were observed in children who did not have sarcopenia at the diagnosis. Comparison of baseline characteristics between children with persistent sarcopenia (*N* = 20) and those who resolved sarcopenia (*N* = 18) is reported in Table S1. Patients with persistent sarcopenia showed significantly lower muscle mass (*z*-score L3-L4 tPMA −2.2 vs −2, *P* = .01). Persistent sarcopenia was associated with more frequent corticosteroid courses during follow-up (65% vs 33%, *P* = .05), despite comparable induction treatments.

## Discussion

This study builds on the current knowledge of the impact of sarcopenia in the management of pediatric patients with CD. To the best of our knowledge, it is the first to focus on the effects of sarcopenia at diagnosis on clinical disease outcomes. We observed that 59% of children in our cohort presented with sarcopenia. This prevalence is notably higher than the 43% and 46% reported in the 2 pediatric studies that employed the same diagnostic methodology.^24^^,^^25^ Our findings revealed that patients with sarcopenia had a significantly higher relapse rate, particularly within the first 6 months (19.5% vs 3%), and a 2-fold increased risk of relapse according to survival analysis. Importantly, sarcopenia emerged as an independent risk factor for clinical relapses, emphasizing the necessity of addressing nutritional deficiencies as part of disease management at diagnosis. However, no significant differences were detected for other clinical outcomes.

While extensive research on sarcopenia exists in adult populations, data in children remain limited. In adults with both CD and ulcerative colitis (UC), sarcopenia has been associated with increased disease activity and adverse clinical outcomes, particularly in the surgical context.^14–16^^,^^26^ Recent studies in 2024 have highlighted growing interest in this field, albeit with conflicting results. Minawala et al.^14^ examined 120 individuals with IBD undergoing intestinal resection and demonstrated that sarcopenia, as measured by SMI, was associated with an increased risk of postoperative complications among older adults. They suggested preoperative SMI assessment via imaging as a valuable tool for risk stratification in this population. Conversely, Donnelly et al.^26^ investigated 124 consecutive CD patients undergoing resection and found that myosteatosis, rather than sarcopenia, was significantly associated with worse postoperative outcomes. In the post-surgical context, a pediatric study focusing on 29 children with UC who underwent colectomy showed that low muscle mass, as measured by MRI of the paraspinous and psoas muscles, was associated with higher postoperative complication rates.^16^ Nonetheless, studies in pediatric populations remain scarce. The largest study in pediatric IBD to date included 101 patients, of whom 69 (68%) had CD, and identified a higher incidence of relapses and an increased likelihood of requiring biologic therapy in patients with sarcopenia.^18^ These results are in line with ours, although the study of Atlan et al.^18^ did not focus on children at the diagnosis but performed a subgroup analysis of 39 children (with both CD and UC) who underwent MR within 3 months of diagnosis, confirming their findings in this small subset of IBD patients. Another study involving 58 CD and 27 UC children evaluated sarcopenia through DXA, analyzing its association with vitamin D status and body composition but found no significant correlations. Recently, a small study of 30 CD children at diagnosis assessed sarcopenia, focusing primarily on the performance of BIA as a cost-effective alternative to MRI. Differently from our results, it did not find an association between the tPMA *z*-score and relapse risk in the first 12 months.^24^ More recently, an Italian study on children with newly diagnosed CD assessed sarcopenia via MRI and found that, although sarcopenic children experienced a higher rate of disease flares during a median 35-month follow-up, no significant differences were observed in terms of composite clinical outcomes, highlighting the need for larger studies to clarify prognostic implications.^25^ Comparison remains hard given the different diagnostic methods used in the various studies, but our findings suggest that sarcopenia might serve as an early marker of disease severity and a predictor of adverse clinical outcomes in pediatric CD.

Regarding the biochemical and metabolic marker analysis in our cohort, we noticed significantly higher total cholesterol and LDL levels in patients with sarcopenia at the diagnosis, which nonetheless fell into the normality range. This difference may be attributed to metabolic dysfunction, chronic inflammation, malnutrition, and reduced physical activity^27^ which could be worse in children with sarcopenia. It is known that there’s a link between chronic inflammation and an altered lipid profile. Pro-inflammatory cytokines such as TNF, interleukin-1 (IL-1), and IL-6 profoundly disrupt lipid metabolism, increasing serum triglycerides and very low-density lipoprotein (VLDL) levels while promoting the formation of small, dense LDL particles. Concurrently, inflammation is associated with decreased HDL levels.^28^ Investigations on larger cohorts could help define whether metabolic blood alterations could serve as a red flag for suspecting the presence of sarcopenia.

Interestingly, our analysis showed that protein and albumin levels were similar in patients with and without sarcopenia, indicating that these biochemical markers may not reliably reflect muscle mass or nutritional status. Albumin, although commonly used to assess nutritional status,^29^ did not identify sarcopenia in our cohort. This finding underscores the distinction between sarcopenia and malnutrition and reflects the complex interplay between muscle mass and nutritional markers. Notably, reductions in visceral protein concentrations such as albumin are often driven by inflammation rather than true malnutrition,^30^^,^^31^ and it is well established that obesity can coexist with low muscle mass, a condition known as sarcopenic obesity.^32^

Regarding the resolution of sarcopenia, this occurred in our cohort in half of the children at 24 months. Of course, the value of these data is limited by the availability of a subsequent MR analysis in only 38 children. The analysis of the baseline characteristics of children with or without persistent sarcopenia revealed an association only with the baseline tPMA values which were significantly lower at diagnosis in patients with persistent sarcopenia. Moreover, a higher need for steroid therapy during follow-up was observed in children who did not experience resolution of sarcopenia, supporting the known catabolic effects of glucocorticoids on muscle. Chronic glucocorticoid exposure accelerates muscle atrophy by disrupting protein homeostasis, specifically by inhibiting protein synthesis through reduced insulin-like growth factor1 (IGF-1) signaling and upregulating proteolytic pathways. Even short-term use can lead to rapid muscle loss, underscoring the importance of steroid-sparing strategies and vigilant monitoring to prevent long-term deficits in muscle mass and function.^33^

Our study has some limitations, inherent to its retrospective design and to the absence of pediatric-specific guidelines for defining sarcopenia. First, the sample size was constrained by the availability of eligible patients, precluding a priori power calculations. While we observed a clinically meaningful difference in relapse rates between children with and without sarcopenia (65% vs 28%), post hoc analysis revealed only moderate statistical power (66% at α = 0.05). This suggests the possibility of type II errors for smaller effect sizes. Additionally, the single-center design limits generalizability. Finally, the high prevalence of sarcopenia observed in our cohort may reflect a selection bias, as MRE could have been more extensively conducted in children with a higher risk of complications and small bowel involvement at diagnosis. Our center’s extensive expertise in capsule endoscopy further influences this bias, as this diagnostic tool is routinely performed in nearly all patients.

Despite these limitations, our findings are somehow strengthened by the sample size, which is the largest pediatric CD cohort at diagnosis, and by the rigorous methodology used to define sarcopenia, with uniform measurement in all children based on the latest recommendations for pediatric reference values.^22^

### Conclusion

Sarcopenia represents a significant yet often underrecognized challenge in the management of pediatric CD. Its presence at diagnosis is associated with a higher risk of relapses, particularly within the first year. A major hurdle in addressing sarcopenia is the lack of standardized diagnostic guidelines for pediatric populations. While limited access to diagnostic tools like DXA and BIA in many clinical settings complicates early identification, the widespread availability of MRI offers a practical alternative. Sarcopenia is well known to affect the quality of life of individuals and their families. Addressing sarcopenia should, therefore, be a therapeutic goal, as its persistence during childhood may have long-term consequences on growth and development.^34^ Nutritional and physiotherapeutic interventions have been shown to promote muscle mass recovery within the first 2 years following a sarcopenia diagnosis, highlighting the importance of a multidisciplinary approach.^12^^,^^35^ Future research should focus on further elucidating the role of body composition in the clinical trajectory of pediatric CD and on defining sarcopenia as a potential negative prognostic marker. In particular, prospective studies are needed to explore the impact of sarcopenia on patient-reported outcomes such as fatigue, which, although beyond the scope of the present study, represents a clinically significant and underrecognized dimension of disease burden. The development of standardized screening and nutritional intervention protocols will be essential to improving clinical outcomes and enhancing the quality of life for affected children.

## Supplementary Material

izaf193_Supplementary_Data

## References

[1] Torres J , Mehandru S, Colombel JF, et al Crohn’s disease. Lancet. 2017;389:1741-1755. 10.1016/S0140-6736(16)31711-1 27914655

[2] Kuenzig ME , Fung SG, Marderfeld L, et al InsightScope Pediatric IBD Epidemiology Group. Twenty-first century trends in the global epidemiology of pediatric-onset inflammatory bowel disease: systematic review. Gastroenterology. 2022;162:1147-1159.e4. 10.1053/j.gastro.2021.12.282 34995526

[3] Sarter H , Crétin T, Savoye G, et al EPIMAD study Group. Incidence, prevalence and clinical presentation of inflammatory bowel diseases in Northern France: a 30-year population-based study. Lancet Reg Health Eur. 2024;47:101097. 10.1016/j.lanepe.2024.101097 39478988 PMC11522416

[4] Bezzio C , Brinch D, Ribaldone DG, et al Prevalence, risk factors and association with clinical outcomes of malnutrition and sarcopenia in inflammatory bowel disease: a prospective study. Nutrients. 2024;16:3983. 10.3390/nu16233983 39683376 PMC11643262

[5] Hartman C , Eliakim R, Shamir R. Nutritional status and nutritional therapy in inflammatory bowel diseases. World J Gastroenterol. 2009;15:2570-2578. 10.3748/wjg.15.2570 19496185 PMC2691486

[6] Heuschkel R , Salvestrini C, Beattie MR, et al Guidelines for the management of growth failure in childhood inflammatory bowel disease. Inflamm Bowel Dis. 2008;14:839-849. 10.1002/ibd.20378 18266237

[7] Aljilani B , Tsintzas K, Jacques M, et al Systematic review: sarcopenia in paediatric inflammatory bowel disease. Clin Nutr ESPEN. 2023;57:647-654. 10.1016/j.clnesp.2023.08.009 37739718

[8] Atia O , Lujan R, Buchuk R, et al Predictors of complicated disease course in adults and children with Crohn’s disease: a nationwide study from the epi-IIRN. Inflamm Bowel Dis. 2024;30:2370-2379. 10.1093/ibd/izae014 38330226

[9] Rosenberg IH. Sarcopenia: origins and clinical relevance. J Nutr. 1997;127:990S-991S. 10.1093/jn/127.5.990S 9164280

[10] Fearon K , Strasser F, Anker SD, et al Definition and classification of cancer cachexia: an international consensus. Lancet Oncol. 2011;12:489-495. 10.1016/S1470-2045(10)70218-7 21296615

[11] Whyte K , Gallagher D. 1.2.3 Technical measurements of body composition assessment. World Rev Nutr Diet. 2022;124:23-30. 10.1159/000517192 35240646 PMC12208704

[12] Sayer AA , Cooper R, Arai H, et al Sarcopenia. Nat Rev Dis Primers. 2024;10:68. 10.1038/s41572-024-00550-w 39300120

[13] Levine A , Koletzko S, Turner D, et al European Society of Pediatric Gastroenterology, Hepatology, and Nutrition. ESPGHAN revised porto criteria for the diagnosis of inflammatory bowel disease in children and adolescents. J Pediatr Gastroenterol Nutr. 2014;58:795-806. 10.1097/MPG.0000000000000239 24231644

[14] Minawala R , Kim M, Delau O, et al Sarcopenia is a risk factor for postoperative complications among older adults with inflammatory bowel disease. Inflamm Bowel Dis. 2025;31:1537-1547. 10.1093/ibd/izae187 39177976 PMC13030995

[15] O’Brien S , Kavanagh RG, Carey BW, et al The impact of sarcopenia and myosteatosis on postoperative outcomes in patients with inflammatory bowel disease. Eur Radiol Exp. 2018;2:37. 10.1186/s41747-018-0072-3 30460523 PMC6246753

[16] Dedhia PH , White Y, Dillman JR, et al Reduced paraspinous muscle area is associated with post-colectomy complications in children with ulcerative colitis. J Pediatr Surg. 2018;53:477-482. 10.1016/j.jpedsurg.2017.09.006 29103786

[17] Zhang T , Cao L, Cao T, et al Prevalence of sarcopenia and its impact on postoperative outcome in patients with Crohn’s disease undergoing bowel resection. J Parenter Enteral Nutr. 2017;41:592-600. 10.1177/0148607115612054 26471990

[18] Atlan L , Cohen S, Shiran S, et al Sarcopenia is a predictor for adverse clinical outcome in pediatric inflammatory bowel disease. J Pediatr Gastroenterol Nutr. 2021;172:883-888. 10.1097/MPG.0000000000003091 33720095

[19] Turner D , Griffiths AM, Walters TD, et al Mathematical weighting of the pediatric Crohn’s disease activity index (PCDAI) and comparison with its other short versions. Inflamm Bowel Dis. 2012;18:55-62. 10.1002/ibd.21649 21351206

[20] Daperno M , D’Haens G, Van Assche G, et al Development and validation of a new, simplified endoscopic activity score for Crohn’s disease: the SES-CD. Gastrointest Endosc. 2004;60:505-512. 10.1016/s0016-5107(04)01878-4 15472670

[21] Levine A , Griffiths A, Markowitz J, et al Pediatric modification of the Montreal classification for inflammatory bowel disease: the Paris classification. Inflamm Bowel Dis. 2011;17:1314-1321. 10.1002/ibd.21493 21560194

[22] Lurz E , Patel H, Lebovic G, et al Paediatric reference values for total psoas muscle area. J Cachexia Sarcopenia Muscle. 2020;11:405-414. 10.1002/jcsm.12514 31920002 PMC7113526

[23] Yamada R , Tsuruta T, Todo Y, et al Validity of skeletal muscle mass index measurements for assessing sarcopenia in patients with gynecological cancer. Jpn J Clin Oncol. 2021;51:1534-1540. 10.1093/jjco/hyab116 34327536

[24] Blagec P , Sara S, Tripalo Batoš A, et al Magnetic resonance imaging can be used to assess sarcopenia in children with newly diagnosed Crohn’s disease. Nutrients. 2023;15:3838. 10.3390/nu15173838 37686870 PMC10490346

[25] Calia M , Rebora P, Gandola D, et al Investigating sarcopenia in pediatric Crohn’s disease with magnetic resonance enterography: an observational study. Clin Nutr ESPEN. 2025;68:14-21. 10.1016/j.clnesp.2025.04.027 40315990

[26] Donnelly M , Driever D, Ryan ÉJ, et al Obesity, sarcopenia and myosteatosis: impact on clinical outcomes in the operative management of Crohn’s disease. Inflamm Bowel Dis. 2024;30:1517-1528. 10.1093/ibd/izad225 37861366 PMC11369076

[27] Mager DR , Carroll MW, Wine E, et al Vitamin D status and risk for sarcopenia in youth with inflammatory bowel diseases. Eur J Clin Nutr. 2018;72:623-626. 10.1038/s41430-018-0105-2 29391593

[28] Al Saedi A , Debruin DA, Hayes A, et al Lipid metabolism in sarcopenia. Bone. 2022;164:116539. 10.1016/j.bone.2022.116539 36007811

[29] Khovidhunkit W , Kim MS, Memon RA, et al Effects of infection and inflammation on lipid and lipoprotein metabolism: mechanisms and consequences to the host. J Lipid Res. 2004;45:1169-1196. 10.1194/jlr.R300019-JLR200 15102878

[30] Evans DC , Corkins MR, Malone A, et al ASPEN Malnutrition Committee. The use of visceral proteins as nutrition markers: an ASPEN position paper. Nutr Clin Pract. 2021;36:22-28. 10.1002/ncp.10588 33125793

[31] Massironi S , Viganò C, Palermo A, et al Inflammation and malnutrition in inflammatory bowel disease. Lancet Gastroenterol Hepatol. 2023;8:579-590. 10.1016/S2468-1253(23)00011-0 36933563

[32] Donini LM , Busetto L, Bischoff SC, et al Definition and diagnostic criteria for sarcopenic obesity: ESPEN and EASO consensus statement. Obes Facts. 2022;15:321-335. 10.1159/000521241 35196654 PMC9210010

[33] Steell L , Gray SR, Russell RK, et al Pathogenesis of musculoskeletal deficits in children and adults with inflamm bowel dis. Nutrients. 2021;13:2899. 10.3390/nu13082899 34445056 PMC8398806

[34] Inoue T , Wakabayashi H, Kawase F, et al Diagnostic criteria, prevalence, and clinical outcomes of pediatric sarcopenia: a scoping review. Clin Nutr. 2024;43:1825-1843. 10.1016/j.clnu.2024.06.024 38959660

[35] Kakehi S , Wakabayashi H, Inuma H, et al Rehabilitation nutrition and exercise therapy for sarcopenia. World J Mens Health. 2022;40:1-10. 10.5534/wjmh.200190 33831974 PMC8761238

