# Supplementary material for: The Impact of Sarcopenia on Clinical Outcomes in Pediatric Crohn’s Disease
Source: Inflamm Bowel Dis. 2025 Sep 5;32(1):67–76. doi: 10.1093/ibd/izaf193 (PMC12759052; doi:10.1093/ibd/izaf193)
Supplement: izaf193_Supplementary_Data [file izaf193_supplementary_data.zip › supplementary table 1_rev1.docx]

| ***Supplementary table 1****. Comparison of baseline clinical parameters and laboratory values between patients with persistent sarcopenia vs. patients without persistent sarcopenia at 24 months follow up* | | | |
| --- | --- | --- | --- |
|  | **Sarcopenia persistent**  **N=20** | **Sarcopenia not persistent**  **N=18** | **p** |
| **Age, median (IQR)** | 11 (9-16) | 9 (8-12.2) | 0.008 |
| **BMI Z-score, median (IQR)** | -0.16 (-0.3 -0.67) | - 0,3 (-1 - -0.02) | 0.01 |
| **Behavior, N (%)**  *B1*  *B2*  *B3* | 18 (90)  2 (10)  0 (0) | 18 (100)  0 (0)  0 (0) | 0.4  0.4  1 |
| **Location, N (%)**  *L1*  *L2*  *L3*  *L4*  *p* | 0 (0)  2 (10)  18 (90)  10 (50)  4 (20) | 2 (11)  0 (0)  16 (89)  10 (56)  4 (22) | 0.2  0.4  1  0.7  1 |
| **Protein (g/L), median (IQR)** | 72 (70.2-77.5) | 72 (70-75) | 0.2 |
| **Albumin (g/L), median (IQR)** | 42 (40.2-44) | 39 (36-39) | 0.003 |
| **RCP (mg/dL), median (IQR)** | 1.2 (0.2-2.1) | 1.3 (1.1-5.1) | 0.03 |
| **ESR (mm/h), median (IQR)** | 47 (21.2-80.7) | 65 (40-85) | 0.058 |
| **wPCDAI, median (IQR)** | 45 (40-52.5) | 35 (13.1-37.5) | 0.0007 |
| **SES-CD, median (IQR)** | 17 (15-20) | 13 (7.5-13.25) | 0.0005 |
| **z-score L3-L4 tPMA, median (IQR)** | -2.2 (-2.7 - -2) | -2 (-2.05 - 2) | 0.01 |
| **Induction treatment, n (%)**  Nutritional therapy (EEN or diet + PEN)  CS  Anti-TNF | 1 (0)  10 (50)  11 (55) | 3 (11)  9 (50)  13 (72) | 0.32  1  0.32 |
| **CS courses during follow up, n (%)** | 13 (65) | 6 (33) | 0.05 |
| *IQR: interquartile range; SD: standard deviation; wPCDAI: weighted pediatric Crohn’s disease activity index; SES-CD: Simple Endoscopic Score for Crohn’s disease; UCEIS: ulcerative colitis endoscopic index of severity; CRP: C-reactive protein; ESR: erythrocyte sedimentation rate, EEN: exclusive enteral nutrition; PEN: partial enteral nutrition; CS: corticosteroids; anti-TNF: anti-tumor necrosis factor.* | | | |
